# Longitudinal studies support the safety and ethics of virtual reality suicide as a research method

**DOI:** 10.1038/s41598-021-89152-0

**Published:** 2021-05-06

**Authors:** Xieyining Huang, Kensie M. Funsch, Esther C. Park, Paul Conway, Joseph C. Franklin, Jessica D. Ribeiro

**Affiliations:** 1 Department of Psychology, Florida State University, 1107 W Call St, Tallahassee, FL 32304 USA; 2 School of Behavioral and Brain Sciences, The University of Texas at Dallas, 800 W. Campbell Rd, Richardson, TX 75080 USA

**Keywords:** Psychology, Human behaviour, Psychiatric disorders

## Abstract

Many have expressed concerns about the safety and ethics of conducting suicide research, especially intense suicide research methods that expose participants to graphic depictions of suicidality. We conducted two studies to evaluate the effects of one such method called virtual reality (VR) suicide. Study 1 tested the effects of VR suicide exposure over the course of one month in participants with (*n* = 56) and without a history of suicidality (*n* = 50). Study 2 exposed some participants to VR suicide scenarios (*n* = 79) and others to control scenarios (*n* = 80). Participants were invited to complete a follow-up assessment after an average of 2 years. For both studies, the presence of suicidality post exposure was the primary outcome, with closely related constructs (e.g., capability for suicide, agitation) as secondary outcomes. Study 1 found no pre-post increases in suicidality or related variables, but revealed several significant *decreases* associated with small to medium effect sizes in suicide-related constructs. In Study 2, VR suicide exposure did not cause any significant increases in suicidality or related variables. Together with prior research, these findings suggest that methods involving intense suicide stimuli appear safe and consistent with utilitarian ethics.

Suicide is an increasingly prevalent public health problem, with a 33% increase in suicide rate in the United States from 1999 to 2017^1^. Given the great urgency to reverse this trend, many national institutions have made repeated calls to understand, predict, and prevent suicide. Despite the push for more research on suicide, there is also resistance to suicide research from some institutions, members of the public, and even researchers^2–6^. Many have expressed concerns that through exposure to suicide-related questions, stimuli, and immersive scenarios, suicide research could potentially cause suicidality. This belief has led some to conclude that certain forms of suicide research—particularly those that involve graphic stimuli—may be unethical. We share these concerns. For us and many other suicide researchers, this prompts a difficult question: *How can we ethically conduct studies to advance knowledge about suicidality?* To address this issue, this paper will consider how ethics relate to suicide research and will present two studies that evaluate the ethics of a particularly intense method of suicide research called virtual reality (VR) suicide.

In many discussions about ethics of suicide research methods, discussants neglect to articulate what they mean by “ethical.” Such articulations are important because ethics is not a monolithic entity. Broadly speaking, there are three main approaches to the study of ethics^7,8^. *Virtue ethics* focus on the moral character of decision-makers across the lifespan^9^, which is unhelpful in determining whether a particular research program is ethical and will not be considered further here. *Deontological ethics* describe ethicality in terms of universal absolute rights and duties, such as the duty to never lie or cause harm^10^. According to deontological ethics, suicide research is unethical if it causes *any* harm regardless of the outcomes it produces. Considering that addressing deontological ethical concerns would require proving a negative (i.e., the absolute absence of any harm), which can be difficult to achieve through scientific inquiries, deontological ethics will not be further discussed here. *Utilitarian* (or more broadly, *consequentialist*) ethics describe the ethicality of actions in terms of outcomes. Actions that result in a net benefit to humanity are deemed morally good even if they involve lying or directly causing a lesser degree of harm than they prevent. Utilitarian ethical systems are commonly adopted by institutional review boards (IRBs) that follow the three broad ethical principles in the Belmont Report: respect for persons, justice, and beneficence^11^. Utilitarian ethics are most evident in the description of beneficence, where it is stated that “benefits and risks must be ‘balanced’ and shown to be ‘in a favorable ratio’” (p. 5).

Following guidelines such as the Belmont Report^11^, we reason that the ethics of suicide research should be evaluated primarily in terms of utilitarian ethics. From this perspective, the potential benefits of suicide research ought to be maximized. Many intensive laboratory-based methods are particularly promising in this domain as they can shed light on processes that self-report assessments alone cannot. Notably, such studies often include procedures such as graphic depictions of suicidality, recollections of suicidal thoughts, negative mood inductions, and physical pain^12–14^. From the utilitarian perspective, it is also important to minimize the potential harms of suicide research. Given the nature of their procedures, laboratory-based methods raise particular concern on this front. To be ethical in a utilitarian sense, a reasonable case must be made that the potential benefits of a given suicide research study outweigh its potential harms.

There are many potential benefits of suicide research, but it is perhaps easiest to evaluate its potential harms. The sum of the evidence indicates that the potential harms of suicide research appear to be indistinguishable from *minimal risk* (i.e., the risk of harm that one might expect in the course of everyday events). A recent meta-analysis found no significant impact of a range of study procedures on suicide ideation and psychological distress after suicidality assessment^15^. Another meta-analysis found that exposure to suicide-related content resulted in a significant though small reduction in suicide ideation and likelihood of engaging in suicidal behaviors^16^. Echoing these findings, Bender and colleagues^17^ showed that higher-risk individuals actually exhibited lower implicit suicidality after suicidality assessment. Many other studies have detected similar patterns^13,18,19^. In terms of utilitarian ethics, evidence indicates that suicide research is ethical because its potential harms appear to be consistent with minimal risk and may be outweighed by its potential benefits (e.g., advancing knowledge, reducing suicidality).

One important caveat to this conclusion, however, is that the procedures employed in most existing laboratory studies of suicidality were of a low-to-moderate intensity. It is possible that more immersive or intense stimuli might cause suicidality or related harms. A recently validated suicide research method called VR suicide exposes participants to particularly vivid and intense suicide-related stimuli^20^. Participants in these studies were placed in realistic, three-dimensional, first-person VR scenarios and given the opportunity to engage in a virtual suicidal behavior (e.g., jumping from a virtual building, virtually shooting oneself). Participants’ virtual suicidal behavior was used as an outcome approximating actual suicide. Initial evidence supports the validity of this, albeit imperfect, approximation^20^. Across three studies with close to 500 undergraduate students as participants, VR suicide scenarios were regarded as realistic and relevant to suicide; VR suicide rates were on par with those of actual suicidal behaviors in the sample (~ 5%); reported reasons for not engaging in VR suicide (e.g., “I am just not the kind of person who would ever choose the virtual suicide option,” “the virtual suicide option was just too scary,” “because of moral/religious reasons”) were akin to those for not engaging in actual suicidal behavior; and the predictors of VR suicide resembled predictors of actual suicide (e.g., suicidal desire, agitation, male sex, a history of suicidal thoughts and behaviors).

This new method has major potential benefits as it is one of the few methods that can shed light on the causes of suicidal behavior, and such causal information is crucial for developing better suicide prevention techniques. For example, a recent experiment adopting this method found that experienced stress alone did not cause an increase in VR suicide, but the anticipation that VR suicide would help avoid stress resulted in an increase in VR suicide^21^. The findings of this study have broad implications for suicide prevention efforts: they suggest that targeting how individuals conceptualize the consequence of suicide may reduce suicide risk. Despite its potential to advance suicide knowledge, the highly vivid and realistic nature of this method raises questions about its potential harms, such as increasing capability for suicide or “triggering” suicidality.

Initial work on the VR suicide method evaluated the immediate impact of multiple VR suicide scenario exposures among a low-risk sample of undergraduate students who were not specifically recruited for mental health symptoms or suicidal thoughts and behaviors^20^. Consistent with the broader literature on the effects on suicide stimuli exposure^15^, there was no immediate (i.e., within-session) effect of VR suicide scenario exposure on suicide ideation or related cognitions, implicit associations with suicide, implicit/explicit affect toward suicide, fearlessness about death, distress, or clinician-rated suicide risk. These findings are encouraging, but they cannot speak to potential effects of VR suicide exposure among people with suicidality or the medium- and long-term effects of VR suicide exposure. This latter limitation dovetails with a major limitation of the broader literature on the effects of suicide stimuli exposure: nearly all studies investigate the impact of exposure in the immediate term (i.e., end of the study session) or short-term (i.e., a few days or a week). Although these timeframes are well-suited to detect effects that are strongest immediately post exposure, such as habituation, sensitization, and priming effects, such timeframes may not be sufficient to detect effects on suicidal behavior, which may only be evidenced several weeks or even years after exposure.

The present investigation aims to address these limitations. Specifically, Study 1 adopted a quasi-experimental approach and tested the medium-term effects of VR suicide exposure over the course of one month. Study 1 also addressed whether VR suicide exposure may be particularly harmful for higher-risk individuals (i.e., with a history of suicidality). Study 2 adopted a randomized experimental design and tested the long-term effects of VR suicide exposure over the course of two years among a low-risk sample. Consistent with the broader literature and Franklin et al.^20^, we hypothesized that both studies would support the safety of VR suicide exposure. That is, we expected that VR suicide exposure would not increase any form of suicidality. Support for the safety of this method would indicate that, even with intense suicide-related study procedures, suicide research is ethical because it carries a low risk for potential harms.

## Study 1

The primary goals of Study 1 were to examine (1) whether exposure to VR suicide scenarios significantly increases suicidality and related harms over a one-month period; and (2) whether VR suicide exposure may be particularly harmful for individuals with a history of suicidality compared with individuals without a history of suicidality. To achieve these two goals, we adopted a pretest–posttest design where we measured suicidality and related constructs among both the control group without a history of suicidality and the group with a history of suicidality before VR suicide exposure and one month after exposure. Consistent with prior evidence^16,17^, we hypothesized that exposure to immersive and intense VR suicide scenarios would not increase suicidality in the medium term. We also hypothesized that VR suicide scenarios would not be more harmful for individuals with a history of suicidality. Instead, it is possible that small reduction in suicidality or related constructs may manifest among at-risk individuals.

## Methods

### Participants

Participants were recruited from a pool of undergraduate university students enrolled in psychology courses who completed a mass screening survey for research participation credit. To address our research questions, we intentionally recruited a control group without a history of suicide ideation or attempt (*n* = 56) and a group with prior suicide ideation or attempt (STB group: *n* = 62). That is, we oversampled for participants with a history of suicidality such that sample sizes would be roughly equivalent between groups. Control group participants were identified by negative responses on two screening items: “Have you ever had thoughts of killing yourself?” and “Have you ever made an actual attempt to kill yourself in which you had at least some intent to die?” Participants who responded negatively to both items were invited to participate in the study as the control group, and those who responded positively to either of the two items were invited to participate as the STB group. Based on participants’ mass screening survey responses, 11 (17.74%) of the 62 participants in the STB group have attempted suicide in their lifetime, and 51 (82.26%) of them have thought about suicide but have not attempted. Participants were young (*M* = 19.56, *SD* = 1.95) and primarily female (79.66%). More than half of the participants self-identified as White/Caucasian (63.56%), with the rest identifying as Hispanic/Latino (16.10%), Black/African-American (13.56%), Asian/Pacific Islander (5.08%), and Mixed or Other Race/Ethnicities (1.69%). The control group and STB group did not differ in terms of demographics.

### Procedure

The IRB at Florida State University approved all study procedures.

#### Baseline

**Figure 1:**
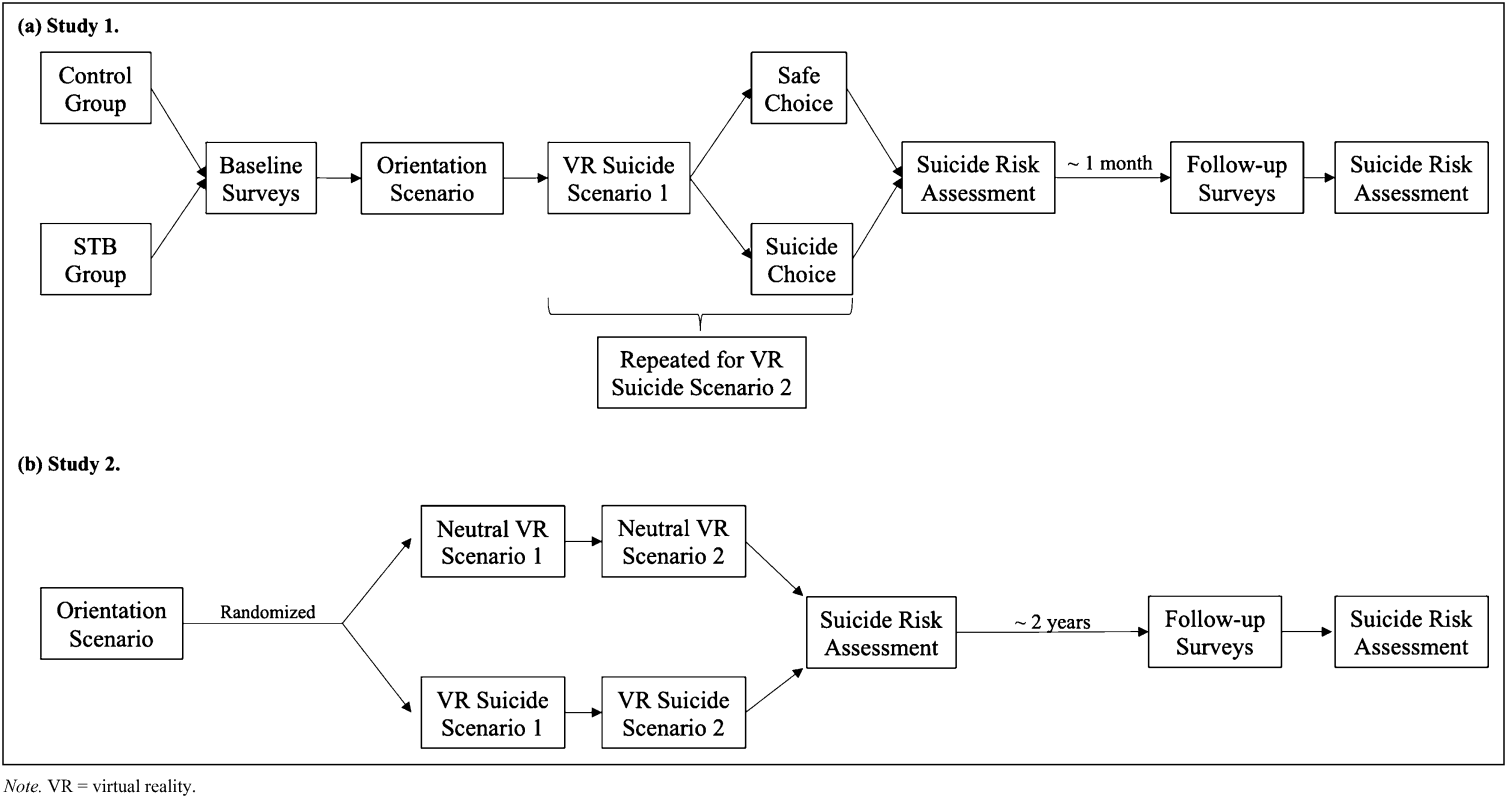
Flow Charts of Study Procedures. *Note.* VR = virtual reality.

We adopted the procedures used by Franklin et al.^20^ (see Study 2) for participants’ first study visit (Fig. 1). Due to space limitation, we provide a brief description here and a more detailed description in Supplements 1 & 2. Briefly, participants read and signed an informed consent form upon arrival. Participants then completed a series of baseline questionnaires. Afterward, they were oriented to the instruction structure for the upcoming virtual reality scenarios and practiced following instructions in a VR orientation scenario. All participants underwent two VR suicide scenarios, the order of which was counterbalanced. In each VR scenario, participants were offered an explicit choice to engage in virtual suicide or a safe alternative. The order of the presentation of the two choices was counterbalanced as well. Lastly, participants experienced a VR scenario as a positive mood induction. All participants were assessed for suicide risk by trained staff based on the framework proposed by Joiner et al.^22,23^; corresponding steps were taken to mitigate risk (see Supplement 1 for details). Participants were then debriefed and compensated for this study visit. The baseline visit lasted approximately 1.5 h.

#### Follow-up assessment

Approximately one month (*M* = 28.39 days, *SD* = 4.03) after the initial study visit, participants returned to the laboratory to complete the follow-up assessment. Participants filled out the same series of questionnaires from baseline, but they were instructed to base their responses on experiences since the last study visit. The follow-up assessment lasted approximately 30 min. Afterward, participants were interviewed by trained staff to complete a suicide risk assessment^22,23^. Corresponding steps were taken to mitigate risk based on their risk level. All participants were then debriefed and compensated with a $50 electronic gift card.

### Measures

#### Demographics

Participants responded to items regarding their sex, age, and race/ethnicity at baseline.

#### Primary outcome measures

Given that we were mainly interested in examining whether intense suicide research stimuli might increase suicidality among participants, the primary outcomes were the presence or absence of suicidal thoughts and behaviors. At baseline, participants answered true/false to the following items, which were designed to assess any experience of suicidal thoughts and behaviors: “I have seriously considered suicide before;” “I have planned a suicide attempt before;” and “I have attempted suicide (i.e., purposefully injured myself with some intent to die) before.” At follow-up, participants answered the same items again, except that the items were modified to inquire about their suicidal thoughts and behaviors since their last study visit (e.g., “I have seriously considered suicide since my last study visit in this lab”). Participants’ clinician-rated suicide risk levels^22,23^ were also examined to investigate whether exposure to VR suicide would lead to clinically meaningful changes that necessitate changes in risk mitigation strategies (see Supplement 1 for detailed procedures).

#### Secondary outcome measures

In addition to investigating whether exposure to intense suicide stimuli increases suicidal thoughts and behaviors, we examined whether such exposure might lead to harm in closely related areas. As hundreds and thousands of risk factors have been found to be significantly yet weakly predictive of future suicidality^24^, we were unable to include all relevant measures. Because the presence of capability for suicide, perceived burdensomeness, and thwarted belongingness is posited to cause suicide according to one of the most popular theories in the field^25,26^, we elected to include these constructs. We also assessed for agitation as it is routinely listed as a suicide warning sign by multiple leading suicide prevention organizations^27,28^. Emotion dysregulation and self-criticism were also assessed given their theoretical relevance to suicidality^29–32^.

#### Acquired capability for suicide scale—fearlessness about death

We adopted one of the gold standard measures in the field, the Acquired Capability for Suicide Scale—Fearlessness about Death (ACSS-FAD), to assess capability for suicide^33^. The ACSS-FAD involves seven items assessing for acquired capability for suicide, a construct posited to increase risk for suicidal behaviors^25,26^. Participants responded to the items, such as “the pain involved in dying frightens me,” by rating on a Likert scale from 0 (not at all like me) to 4 (very much like me). The internal consistency was good at baseline (*α* = 0.84) and at follow-up (*α* = 0.84).

#### Interpersonal needs questionnaire (INQ-15)

The INQ-15 was adopted to assess perceived burdensomeness and thwarted belongingness^34^, two constructs that have been proposed to lead to suicidal desire^25,26^. The perceived burdensomeness subscale includes six items, and the thwarted belongingness subscale includes nine items. Each item (e.g., “these days, I think I am a burden on society”) was rated on a scale of 1 (not at all true for me) to 7 (very true for me). The questionnaire has shown good psychometric properties, such as good reliability, and convergent and divergent validity. Both subscales demonstrated good internal consistency across two timepoints (*α*s = 0.87—0.92).

#### Brief agitation measure (BAM)

The BAM is a three-item measure that assesses current agitation levels^35^. Each item was rated on a scale of 0 (strongly disagree) to 6 (strongly agree). Cronbach’s alpha ranged from 0.86 at baseline to 0.90 at follow-up.

#### Difficulties in emotion regulation Scale (DERS)

The DERS is a 36-item scale assessing emotional dysregulation^36^. Participants rated each item (e.g., “I am clear about my feelings”) on a scale of 1 (almost never) to 5 (almost always). The DERS has demonstrated good test–retest reliability^36^, high internal consistency^37–39^, as well as good construct and predictive validity^40,41^. The internal consistency within this study was good at both timepoints (baseline: *α* = 0.95; follow-up: *α* = 0.96).

#### Self-rating scale (SRS)

Participants completed the SRS^42^ to assess their self-critical beliefs. Participants rated each of the eight items such as “I am deserving of pain and punishment” on a Likert scale ranging from 1 (strongly disagree) to 7 (strongly agree). The SRS has demonstrated strong internal reliability^42,43^. Within the study, Cronbach’s alpha ranged from 0.89 at baseline to 0.93 at follow-up.

### Data analytic plan

All data analyses were performed using base R version 4.0.0^44^. Given that the sample size of participants with a history of suicide attempt was small (*n* = 11), we analyzed data from participants with a history of suicide ideation and suicide attempt together (i.e., the STB group). We also conducted analyses based on three distinct groups (i.e., control group, suicide ideation group, suicide attempt group); results were largely consistent with the exception that power substantially decreased among the suicide attempt group. As such, we reported analytic results from comparing the control group with the STB group in the main text and included analytic results from comparing the three groups in Supplement 3.

Considering that our research goals were to study (1) potential medium-term harm post VR suicide exposure and (2) potential exacerbation in harm for individuals with a history of suicidality, our main analyses involved (1) examining pre-post effects of experiencing VR suicide scenarios within each group and (2) testing for interaction effects between exposure to VR suicide scenarios and group membership (i.e., time [pre, post] × group [control, STB] interaction). That is, we examined whether the pre-post effects of exposure to VR suicide scenarios were different depending on the group (e.g., whether the STB group might experience much larger negative consequences after exposure than the control group). Given that it is possible that iatrogenic effects might only be present for individuals who engaged in virtual suicide, we additionally compared the follow-up measure scores of participants who engaged in virtual suicide with those who did not engage in virtual suicide. Independent samples and paired *t*-tests were adopted to investigate the effects of exposure to VR suicide scenarios on continuous measures. Chi-squared analyses and Fisher’s exact tests were adopted for categorical outcomes. Of note, we compared between-group differences at each timepoint. As these analyses were less essential than our main analyses given our research aims, the results were reported in Supplement 4.

Multiple strategies were used to address missing data and the associated potential of biases. We first conducted analyses based on complete cases. We then adopted multiple imputation using the *r* package *mice* version 3.9.0^45^. Five imputed datasets were created, and results were averaged. As findings were consistent across the two approaches, we reported results from complete cases in the main text and results from multiple imputations in Supplement 5. Considering the longitudinal nature of the study, participants who dropped out might significantly differ from those completing the follow-up assessment. To control for potential differences, regression analyses were conducted to obtain adjusted effects of exposure to VR suicide scenarios. For each regression analysis examining pre-post differences within each group, the dependent variable was the outcome of interest (e.g., agitation), and the independent variable was the outcome of interest assessed at baseline. Differences between the retained sample and participants lost to follow-up were entered into the regression model as covariates. A regression coefficient of one for the outcome of interest assessed at baseline indicates no change in the outcome of interest from baseline to follow-up, with coefficients significantly smaller or larger than one indicating significant increases or decreases from baseline. For regression analyses examining differences in pre-post changes by group, the change in scores was considered the dependent variable with group membership as the independent variable. For binary outcomes (e.g., suicide attempt), logistic regression analyses were employed.

## Results

### Retention & missing data

Twelve participants, six in each group, were lost to follow-up, resulting in an overall retention rate of 89.83%. The control group and STB group did not differ in terms of retention rates (control: 89.29%; STB: 90.32%, *χ*^2^ < 0.001, *p* = 0.99). Participants lost to follow-up were significantly younger (lost to follow-up: *M* = 18.83, *SD* = 0.83; retained: *M* = 19.64, *SD* = 2.03; *t* = − 2.60, *p* = 0.01), and scored significantly higher on the DERS (lost to follow-up: *M* = 98.00, *SD* = 24.27; retained: *M* = 81.88, *SD* = 23.67; *t* = 2.19, *p* = 0.047). Participants lost to follow-up did not differ from those who completed the assessment on any other demographic characteristics or baseline measures, including suicide risk. Post-hoc power analyses indicated that there was sufficient power (i.e., 0.80) with the retained sample to detect small effects for continuous outcomes (i.e., Cohen’s *d* ~ 0.2) and medium effects for binary outcomes (i.e., Cohen’s *h* ~ 0.5). For baseline, only one participant’s data were missing on the DERS items. For follow-up, no additional data were missing other than those lost to follow-up.

### Pre-post changes

Neither the control group nor the STB group experienced any worsening in suicidality as measured by self-reported serious suicide ideation, plan, and attempt as well as clinician-rated risk categories (Table 1). Similarly, neither group experienced any increase on related clinical measures (Table 1). Notably, multiple decreases in scores were observed. Both groups reported significantly lower levels of agitation (*p*s < 0.001), emotion dysregulation (*p*s = 0.04), and self-criticism (control: *p* = 0.02; STB: *p* = 0.01). The control group scored significantly lower on capability for suicide (*p* < 0.001), and the STB group reported lower levels of thwarted belongingness (*p* < 0.001) at follow-up (Table 1). These decreases were considered medium to large (*d*s =  − 0.73, − 0.54, respectively). After adjusting for differences between those who dropped out and those who did not (i.e., age and DERS at baseline), both groups continued to show significant decreases in agitation (control: *B* = 0.47, *SE* = 0.10, *p* < 0.001; STB: *B* = 0.54, *SE* = 0.15, *p* < 0.001) and self-criticism (control: *B* = 0.78, *SE* = 0.12, *p* = 0.04; STB: *B* = 0.66, *SE* = 0.12, *p* = 0.004). The decreases in emotion dysregulation in the control group (*B* = 0.74, *SE* = 0.08, *p* = 0.001) and thwarted belongingness in the STB group (*B* = 0.57, *SE* = 0.12, *p* < 0.001) remained significant after considering the covariates.

**Table 1:** Study 1 results.

Measures	Control Group (Baseline: N = 56) (Follow-up: N = 50)				STB Group (Baseline: N = 62) (Follow-up: N = 56)				Between-Group Differences of Pre-Post Changes	
	Mean	SD	Pre-Post		Mean	SD	Pre-Post			
	n	%	t	*p*	n	%	t/χ^2^	*p*	t	*p*
**Primary Outcomes**										
*Serious Suicide Ideation*										
Pre	0	0%			38	61.29%				
One Month Post Exposure	0	0%	–	–	2	3.57%	26.28	** < .001**		
*Suicide Plan*										
Pre	0	0%			14	22.58%				
One Month Post Exposure	0	0%	–	–	0	0%	11.08	**.001**		
*Suicide Attempt*										
Pre	0	0%			11	17.74%				
One Month Post Exposure	0	0%	–	–	0	0%	8.10	**.004**		
*Suicide Risk Category*										
Immediately Post Exposure										
Low	56	100%			51	82.25%				
Low-Moderate	0	0%			11	17.74%				
One Month Post Exposure										
Low	50	100%			49	87.50%				
Low-Moderate	0	0%	–	–	7	12.50%	2.25	.13		
**Secondary Outcomes**										
*ACSS-FAD*										
Pre	11.09	6.07			11.00	6.23				
One Month Post Exposure	9.34	6.70	− 3.57	** < .001**	11.21	6.34	0.72	.47	**2.94**	**.004**^**a**^
*BAM*										
Pre	4.27	4.39			8.15	4.74				
ne Month Post Exposure	2.20	3.04	− 3.71	** < .001**	5.77	5.23	− 3.79	** < .001**	− 0.95	.35
*DERS*										
Pre	71.70	20.31			94.39	22.30				
One Month Post Exposure	65.76	17.26	− 2.06	**.04**	89.09	24.49	− 2.08	**.04**	− 0.40	.69
*INQ—Perceived Burdensomeness*										
Pre	7.07	2.33			11.13	5.39				
One Month Post Exposure	6.68	1.46	− 0.84	.40	10.16	5.28	− 1.81	.08	− 1.81	.19
*INQ—Thwarted Belongingness*										
Pre	18.68	8.68			26.58	8.18				
One Month Post Exposure	17.20	8.46	− 1.28	.21	22.21	8.3	− 4.37	** < .001**	− 2.65	**.009**
*SRS*										
Pre	18.46	9.35			28.58	10.64				
One Month Post Exposure	15.34	9.2	− 2.32	**.02**	25.09	11.81	− 2.68	**.01**	− 0.79	.43

^a^Remained significant after controlling for baseline differences between participants who completed follow-up assessment and those lost to follow-up.

^b^Fisher’s exact test; ACSS-FAD = Acquired Capability for Suicide Scale—Fearlessness about Death; BAM = Brief Agitation Measure; DERS = Difficulties in Emotion Regulation Scale; INQ = Interpersonal Needs Questionnaire; PB = Perceived Burdensomeness; TB = Thwarted Belongingness; SRS = Self-Rating Scale; STB = suicidal thoughts and behaviors.

### Between-group differences

The control and STB groups’ pre-post changes on clinical measures significantly differed on two measures (Table 1). That is, interaction effects between VR suicide exposure and sample severity were detected. First, the control group’s capability for suicide significantly decreased since baseline, whereas the STB group’s capability for suicide remained consistent since baseline (*p* = 0.004). Second, the STB group reported significantly lower levels of thwarted belongingness since baseline but the control group reported similar levels since baseline (*p* = 0.009). These effects were considered small to medium (*d*s = − 0.28, − 0.51, respectively). After adjusting for baseline differences between participants who completed follow-up and those who did not, the between-group differences of pre-post changes on capability for suicide (*B* = 2.47, *SE* = 0.82, *p* = 0.003) remained significant, but not thwarted belongingness (*B* = − 2.81, *SE* = 1.51, *p* = 0.07).

### Effects of engaging in VR suicide

Regardless of whether one engaged in VR suicide, no worsening in suicidality or clinical symptoms after exposure was observed (Supplement 6). In fact, individuals who did not engage in VR suicide experienced significant decreases on the BAM, DERS, INQ-Thwarted Belongingness, and SRS; the effects were consistent after adjusting for age and DERS at baseline (Supplement 6).

## Study 1 discussion

Consistent with our hypothesis and previous research evidence, exposure to VR suicide scenarios did not appear to cause suicidality or related harms among healthy individuals or individuals with a history of suicidality. Instead, both groups experienced small to medium reduction in certain symptoms. Analyses comparing participants who engaged in VR suicide with those who did not also failed to reveal any significant negative consequences. Given that the retained sample size was sufficiently powered to detect medium effects (Cohen’s *h* ~ 0.50) for binary outcomes (e.g., suicide ideation), the current findings do not provide evidence for medium or large iatrogenic effects of VR suicide on suicidality in the medium term, even among higher-risk individuals. However, this study leaves open the question of longer-term effects. Moreover, due to the nature of the pretest–posttest study design, further evidence is needed to shed light on the potential harm and benefits *caused* by this intense suicide research method.

## Study 2

Study 2 aims to test whether exposure to VR suicide might *cause* long-term increase in suicidality and related harm. To achieve this aim and to overcome one limitation of Study 1 (i.e., inability to infer cause), we adopted an experimental design where participants were randomly assigned to either experience suicide or neutral VR scenarios. Based on broader evidence^15,20^ and results from Study 1, we hypothesized that the results would not provide evidence for harm associated with experiencing VR suicide scenarios.

## Methods

### Participants

The 287 participants who took part in the initial VR suicide validation study in 2017 (see Study 1 in Franklin et al.^20^) were retrospectively contacted to complete a follow-up assessment approximately two years later (*M* = 1.75 years; *SD* = 57.78 days). More than half of the participants (*n* = 159; 55.40%) completed the assessment. Among those who participated in the follow-up study, 64.15% were female. Most participants identified their race as White/Caucasian (69.18%), followed by Hispanic/Latino (17.61%), Black/African-American (6.29%), Asian/Asian American (5.66%), Native American (0.63%), and Mixed Race/Ethnicities (0.63%). At the time of follow-up, the average age was 20.41 (*SD* = 1.67). Of note, participants in Study 2 were independent from participants in Study 1.

### Procedure

The IRB at Florida State University approved all study procedures of the follow-up study, including the procedure to contact participants who participated in the initial VR suicide validation study in 2017 and the procedure to compensate participants for the follow-up study.

#### Baseline

A full description of the procedures at baseline may be found in Franklin et al.^20^; we hereby provide a brief description in the main text (Fig. 1) and more detailed description in Supplements 1 & 2. A total of 287 participants were recruited from undergraduate psychology courses. After presenting in the laboratory, participants read and signed an informed consent form. They received an orientation to the VR equipment and experienced an orientation VR scenario. Participants were then randomized to experience either two neutral VR scenarios (*n* = 139) or two VR suicide scenarios (*n* = 148). The order of the scenarios was counterbalanced. Participants rated their experiences in each scenario immediately afterward. At the end, all participants underwent a positive mood induction, a full suicide risk assessment^22,23^, and were debriefed and compensated with course credits. Of note, unlike Study 1 where participants were given the choice of either engaging in suicide or a safe alternative in the suicide scenarios, participants in Study 2 were not provided with a safe alternative. However, they were reminded throughout the study that they could stop participation at any time (see Supplement 2 for detailed experimenter instructions). Because of this difference, approximately 77.70% of the participants randomized to the VR suicide condition engaged in virtual suicide in at least one of the scenarios, with 50% engaging in virtual suicide in both scenarios.

#### Follow-up assessment

Participants were invited to complete a follow-up study via email, which included an electronic link to the survey and their unique ID number from the initial study. The survey lasted approximately 10 min. Because participants were unaware at their initial study visit that a follow-up study would be conducted, a $20 Amazon gift card was sent to participants within 24 h of completion with the goal to increase retention rates. To ensure participants’ safety, participants were required to submit a contact phone number so that, in the event they reported a suicide-related event since participation, they could be contacted by trained researchers to conduct a full suicide risk assessment and take steps to mitigate risk. The same risk assessment procedure was adopted as Study 1^22,23^. Participants who reported any suicide ideation, plan, and attempt since the initial study were contacted by the research staff by phone within 24 h of survey completion. For participants that we were unable to reach upon first attempt, voice messages were left requesting them to call back as soon as possible. Two additional contact attempts were made within 24 h of the first phone message. Due to the voluntary nature of this assessment, we were unable to reach two participants from the control group and five participants from the virtual reality suicide group who needed an assessment; the rates did not differ based on condition. Of note, all participants were provided with mental health resources on the survey website regardless of whether they needed or completed a risk assessment.

### Measures

Similar to Study 1, we were primarily interested in the effects of exposure to VR suicide on suicide ideation, plan, attempt, and risk level since their participation in the initial study. Closely related theoretical constructs such as capability for suicide, thwarted belongingness, and perceived burdensomeness were also examined. The following measures were identical to those administered in Study 1: demographics, ACSS-FAD^33^ (*α* = 0.87), INQ^34^ (thwarted belongingness subscale: *α* = 0.89; perceived burdensomeness subscale *α* = 0.92), and items assessing for the presence of suicide ideation, plan, and attempt since the last study visit. To complement the secondary outcome measures in Study 1, we elected to include a measure on broad psychological symptoms (see below). Again, effects immediately post exposure were previously reported^20^ and therefore not a focus of the present study.

#### Brief symptom inventory 18 (BSI)

The BSI is a self-report rating questionnaire with 18 items designed to assess for psychological symptoms^46^. Participants were instructed to rate how much they had been distressed by each symptom (e.g., faintness or dizziness, feeling lonely) since their participation in the initial study in 2017 on a scale of 1 (not at all) to 5 (extremely). The BSI yields three subscale scores (i.e., depression, anxiety, and somatization) as well as a total score. Higher scores indicate more severe symptoms. The BSI has been shown to demonstrate good psychometric properties^47^. The internal consistency was good (*α* = 0.95).

### Data analytic plan

All data analyses were performed using base R version 4.0.0^44^. First, we examined the unadjusted causal effects of experiencing VR suicide scenarios. Chi-squared analyses and Fisher’s exact tests were employed to examine whether experiencing VR suicide scenarios causes an increase in suicide ideation, plan, attempt, and risk. Independent samples *t*-tests were adopted to investigate the effects of VR suicide scenarios on psychological symptoms, fearlessness of death, perceived burdensomeness, and thwarted belongingness. Second, we examined the adjusted causal effects of experiencing VR suicide scenarios through regression analyses controlling for any potential differences between participants who completed follow-up assessment and those lost to follow-up. The regression models were built in a similar fashion as in Study 1. Lastly, we examined the unadjusted and adjusted effects of engaging in virtual suicide (as opposed to simply experiencing VR suicide scenarios without engaging in virtual suicide). Of note, because the baseline study (i.e., Study 1 in Franklin et al.^20^) was originally designed to evaluate the validity and feasibility of the VR suicide approach, measures on suicidality and related clinical measures were not administered prior to exposure to VR scenarios. Therefore, we were unable compare participants’ scores on measures at the follow-up to their pre-exposure scores. Missing data were addressed in the same way as Study 1. Results based on complete cases were reported in the main text, and results based on multiple imputation were reported in Supplement 5 as they were highly consistent.

## Results

### Retention & missing data

Approximately 57.55% of the participants in the control condition and 53.38% in the suicide VR condition completed the follow-up assessment. The retention rates were not significantly different by condition (*χ*^2^ = 0.35, *p* = 0.55). Among participants assigned to the suicide VR condition, retention rates did not differ between those who engaged in virtual suicide and those who did not (*χ*^2^ = 0.12, *p* = 0.73). Participants lost to follow-up did not differ from those retained at follow-up on suicide risk assessed at the end of the initial study visit (Fisher’s exact test: *p* = 0.99). However, a significantly smaller proportion of Black/African-American participants was retained for follow-up (33.33%; *χ*^2^ = 5.56, *p* = 0.02); and a significantly higher proportion of Asian/Asian American participants was retained (100%; Fisher’s exact test: *p* = 0.005). No other demographic differences were detected. Post-hoc power analyses indicated that the retained sample size would allow for sufficient power (i.e., 0.80) to detect small effects for continuous outcomes (i.e., Cohen’s *d* ~ 0.2) and medium effects for binary outcomes (i.e., Cohen’s *h* ~ 0.5). Other than participants who were lost of follow-up, missing data were only present for one participant on ACSS-FAD and INQ items.

### Effects of exposure to VR suicide scenarios

Previous research has found no immediate (i.e., within-session) effects on safety-related measures after exposure (see Study 1 in Franklin et al.^20^). As such, here we focus on reporting the long-term effects. Overall, the control group participants and the VR suicide group participants did not differ on any safety measures (all *p*s > 0.40; Table 2). Individuals who engaged in VR suicide did not differ on any measures compared with those who did not, with the exception that the former group reported significantly lower levels of anxiety symptoms as measured by the BSI (*p* = 0.046; Supplement 6). Regression analyses adjusting for the effects of race found no significant effects of either exposure to VR suicide scenarios or engaging in virtual suicide.

**Table 2:** Study 2 results.

	Control		Virtual reality suicide		t	p
	(Baseline: N = 139)		(Baseline: N = 148)			
	(Follow-Up: N = 80)		(Follow-Up: N = 79)			
	Mean	SD	Mean	SD		
	n	%	n	%		
**Baseline (post virtual reality scenarios)**						
*Suicide Risk Category*						
No Assessment Needed	125	89.93%	124	83.78%		
Assessment Needed—Low Risk	12	8.63%	23	15.54%		
Assessment Needed—Low-Moderate Risk	2	1.44%	1	0.68%	–	.54^a^
**Follow-Up**						
ACSS-FAD	20.30	7.33	19.59	6.52	− 0.64	.52
BSI	25.20	8.89	24.71	7.97	− 0.36	.72
Somatization	7.38	2.24	7.51	2.21	0.37	.71
Depression	9.12	3.8	8.77	3.83	− 0.57	.57
Anxiety	8.66	3.44	8.56	3.43	− 0.19	.85
INQ—Perceived Burdensomeness	7.01	2.23	6.90	2.52	− 0.28	.78
INQ—Thwarted Belongingness	19.04	10.66	18.05	9.47	− 0.62	.54
Suicide Ideation	2	2.50%	4	5.06%	–	.44
Suicide Plan	0	0.00%	0	0.00%	–	–
Suicide Attempt	0	0.00%	0	0.00%	–	–
*Suicide Risk Category*						
No Assessment Needed	75	93.75%	72	91.14%		
Assessment Needed—Low Risk	3	3.75%	2	2.53%		
Assessment Needed—No Response	2	2.50%	5	6.33%	–	.59^a^

^a^Fisher’s exact test; ACSS-FAD = Acquired Capability for Suicide Scale—Fearlessness about Death; BSI = Brief Symptom Inventory; INQ = Interpersonal Needs Questionnaire; PB = Perceived Burdensomeness; TB = Thwarted Belongingness.

## Study 2 discussion

Results suggest that the VR suicide method did not cause medium or large long-term negative consequences on suicidality and did not cause small to large negative effects on related measures. The findings are consistent with previous studies finding no immediate effects post exposure to VR suicide scenarios^20^ and broader evidence on the lack of iatrogenic effects of exposure to suicide-related content^15,16^.

## General discussion

The present series of studies found no evidence of medium-to-large negative consequences on suicidality from exposure to immersive and intense suicide-related stimuli in the medium term or long term. In addition to not causing harm, the exposure was associated with small-to-medium reduction in certain symptoms for both healthy and at-risk individuals. Given the highly immersive and intense nature of VR suicide scenarios, the results suggest that similar or less intense suicide-related stimuli are unlikely to exert iatrogenic effects in the medium or long term. Together, the findings indicate that—in terms of utilitarian ethics—suicide research adopting these intense methods is ethical because it has the potential for many benefits and carries a low risk for potential harms.

The current findings may seem surprising to some as they contradict two popular—but unsupported—beliefs about the causes of suicidality. The first belief is that suicide-related stimuli cause people to habituate to the pain/fear involved in killing oneself, thereby increasing the capability for suicide (with *capability* being defined as fearlessness about death and the pain involved in dying^25,26^). From this perspective, one might hypothesize that VR suicide would cause suicidality by increasing individuals’ capability for suicide. Although the construct of suicidal capability is widespread in suicide research, it is not supported by empirical evidence: (a) capability for suicide does not appear necessary for suicidal behaviors as it discriminates poorly between individuals with and without suicide attempts cross-sectionally^48,49^ and prospectively^50^; and (b) capability for suicide remains unchanged after self-injury and suicide attempts^51^ and prolonged combat exposure^52^. That is, capability for suicide exerts weak effects on suicidal behaviors, and *actual* suicidal behaviors and other painful and provocative events do not seem to increase capability. As such, it is unlikely that *virtual* suicidal behaviors might significantly increase participants’ capability for suicide. Moreover, even if VR suicide exposure increased the construct of suicidal capability, it would be unlikely to increase risk for suicidality because this construct is weakly associated with suicidality. Consistent with these lines of evidence, no increase in capability for suicide or suicidal thoughts and behaviors were observed immediately after exposure to VR suicide scenarios^20^, or at one-month and two-year follow-up.

The second commonly held belief is that exposure to suicide-related content might “trigger” suicidality. Evidence does not support this belief. A plethora of studies have demonstrated that exposing participants to suicide-related content does not cause negative impacts on self-injurious or suicidal urges^18^, suicide risk^13^, suicide ideation^19,53^, or depressive symptoms^54^. Moreover, multiple studies have shown potential benefits of exposure to suicide-related stimuli, such as reduction in distress, suicide ideation, and depressive symptoms^16,17,55^. Broader evidence outside the field of suicide research also does not support deleterious psychological consequences of exposure to provocative content; in fact, warnings about such content potentially inducing negative reactions may exert countertherapeutic effects^56,57^. In line with previous studies, the current findings indicate that exposure to immersive and intense suicide-related content not only does not cause harm but might be associated with symptom reductions in the medium and long term.

What do the current findings mean regarding the ethics of suicide research adopting these methods? In terms of utilitarian ethics, VR suicide exposure appears to pose minimal risk while providing a potential avenue for scientific advances. However, utilitarian judgments are never static—it is possible that one day evidence will emerge indicating that the costs outweigh the benefits and/or that the cost–benefit ratio is favorable in some situations but not others. For instance, it is possible that certain modifications to the paradigm may improve its construct validity (e.g., incorporating haptic suits to mimic physical pain as participants engage in VR suicide). However, modifications that more closely approximate actual suicidal behavior may increase risk of harm. Therefore, future studies are needed to assess the validity and safety of any novel or updated methods.

It is important to note that the current study findings do not mean that *any* kind of exposure to *any* suicide content is safe and ethical. The current manuscript addresses using suicide-related content for research purpose. In research settings, participants’ suicide risk is monitored, and procedures are in place to mitigate risk. A distinct difference exists between exposure to suicide content in a controlled environment and exposure to any types of suicide content in an uncontrolled environment. Correlational evidence suggests that exposure to certain media portrayal of suicide may be harmful^58^. It is recommended that any portrayal of suicide should follow the responsible reporting guidelines proposed by major health organizations^59,60^.

The study findings need to be considered in the context of its limitations. First, the two samples were overall at low risk for suicide. Because the VR suicide method was originally developed as a translational approach to shed light on basic causal processes present among low-risk populations (e.g., what causes a healthy participant to engage in virtual suicide)^21^, it was not designed with the ultimate goal to place individuals at high or imminent risk for suicide in the virtual suicide scenarios^20^. Translational findings from low-risk samples will require future studies (and likely other types of studies such as observational and longitudinal designs) to be translated to the actual phenomena of interest (e.g., what causes a high-risk patient to die by suicide). If researchers wish to implement the VR suicide method among higher-risk populations, such as individuals with multiple suicide attempts, additional studies are needed to demonstrate its safety. Similarly, this method was initially validated in a primarily young population, and empirical studies are needed to extend the application among older populations.

Second, it is possible that exposure to intense suicide stimuli increased suicide risk and resulted in related harms, but the included measures were not sensitive enough to detect the changes. We reason, however, that this may be implausible. We specifically selected measures that are considered standard and have been widely used in the field with the intention that they may facilitate comparison of the current findings with previous research. Within the present investigation, the measures did appear sensitive enough to reflect pre-post changes in one month: significant *decreases* in capability for suicide, perceived burdensomeness, agitation, and self-criticism were detected in at least one group in Study 1. Therefore, it is unlikely that the lack of observed changes two years post exposure in Study 2 were due to insensitivity of the measures. Moreover, the mechanisms commonly considered to lead to increased risk for suicide after exposure to suicide stimuli (e.g., habituation, sensitization, and priming) typically exert the strongest influence immediately after exposure. Prior studies did not detect increased suicide risk or related harms within session or within days of exposure^13,18^. Given that it is possible that effects on relatively rare phenomena such as suicide ideation and attempt may require a longer period to evidence, the present study extends prior research by examining short- and long-term effects. Consistent with previous studies with longer follow-ups^19^, we found no evidence of exposure to intense suicide stimuli causing suicide ideation, attempt, or related harms in one month and two years.

It is important to note that the current findings do not suggest that VR suicide exposure has *no* effects in *any* domain. Rather, the evidence suggests that it does not appear to cause deleterious effects on suicide risk or related constructs *as currently understood in the literature*. For instance, the exact timeframe needed for one to develop capability for suicide (and relatedly, how much capability for suicide is dangerous) has yet to be clearly delineated. Although capability for suicide was originally theorized to monotonically increase with exposure to painful and provocative events^26^, studies have found contradicting patterns^52,61^. This confusion is compounded by the fact that there lacks research quantifying the sensitivity of measures widely used for these constructs. As more clarity develops on these issues, future research is encouraged to design their studies and update their measures accordingly. Risk and benefits should be re-evaluated as new evidence emerges.

Third, the retained sample sizes for both studies may be small. The retention rate for Study 2 was low despite efforts to increase retention. On balance, the retained sample sizes at follow-up for both Study 1 and Study 2 still had sufficient power for small effects on continuous measures and medium effects on binary outcomes, and Study 1 did detect significant decreases in constructs such as capability for suicide, perceived burdensomeness, and self-criticism. Given that larger samples allow for more precise estimates, future studies are encouraged to test the effects of exposure to intensive suicide-related stimuli with larger sample sizes.

Fourth, we were unaware of any adverse suicide-related outcomes (e.g., suicide death) reported either to us or the IRB, but we were unable to ascertain whether participant dropout was due to suicide ideation, nonfatal suicide attempt, or suicide death. On balance, retention rates were high in Study 1 and statistically equivalent across groups in both Study 1 and Study 2. Additionally, participants retained at follow-up did not differ from those lost to follow-up in terms of suicide risk at the initial study visit. Thus, the potential of this issue significantly impacting findings is likely low. Nonetheless, future studies are encouraged to account for the possibility of participant dropout due to suicidal thoughts and behaviors. Lastly, systematic differences in retained participants were detected. Although analyses controlling for these differences (i.e., age and emotion dysregulation in Study 1, race in Study 2) showed consistent results, future studies are recommended to increase efforts in retaining participants from underrepresented populations and participants who might be more emotionally dysregulated.

In sum, the present investigation did not detect iatrogenic effects of an intense suicide research method over the course of one month, two years, or in higher risk populations. Under the utilitarian system adopted by most scientific institutions, intense suicide research methods such as VR suicide appear to be safe and ethical. Future work should apply such methods to advance knowledge about suicidality while continuing to evaluate the costs and benefits of such research.

## Supplementary Information

Supplementary information.

## References

[1] Hedegaard, H., Curtin, S. & Warner, M. *Suicide mortality in the United States, 1999–2017.* (2018). 30500324

[2] Pearson JL, Stanley B, King CA, Fisher CB (2001). Intervention research with persons at high risk for suicidality: Safety and ethical considerations. J. Clin. Psychiatry.

[3] Lakeman R, Fitzgerald M (2009). Ethical suicide research: A survey of researchers. Int. J. Ment. Health Nurs..

[4] Lakeman R, FitzGerald M (2009). The ethics of suicide research: The views of ethics committee members. Crisis.

[5] Moore M, Maple M, Mitchell AM, Cerel J (2013). Challenges and opportunities for suicide bereavement research: The experience of ethical board review. Crisis.

[6] Andriessen K (2019). Ethical concerns in suicide research: Results of an international researcher survey. J. Empir. Res. Hum. Res. Ethics.

[7] Kagan, S. *Normative ethics*. (Westview Press, 1998).

[8] Hursthouse, R. & Pettigrove, G. Virtue ehics. in *The Stanford encyclopedia of philosophy* (Stanford University, 2018).

[9] Ameriks, K. & Clarke, D. *Aristotle: Nicomachean ethics.* (Cambridge University Press, 2000).

[10] Kant, I. *Foundations of the metaphysics of morals* (L.W. Beck, Trans.). (Bobbs-Merrill, (Original work published 1785), 1959).

[11] National Commission for the Protection of Human Subjects of Biomedical and Behavioral Research. *The Belmont report: Ethical principles and guidelines for the protection of human subjects of research.* https://www.hhs.gov/ohrp/regulations-and-policy/belmont-report/read-the-belmont-report/index.html (1979). 25951677

[12] Orbach I, Mikulincer M, King R, Cohen D, Stein D (1997). Thresholds and tolerance of physical pain in suicidal and nonsuicidal adolescents. J. Consult. Clin. Psychol..

[13] Poindexter EK, Nazem S, Barnes SM, Hostetter TA, Smith PN (2019). Veteran participation in intensive suicide research protocols: No evidence of iatrogenic effects. Suicide Life-Threatening Behav..

[14] Tucker RP, Wingate LRR, Burkley M, Wells TT (2018). Implicit association with suicide as measured by the suicide affect misattribution procedure (S-AMP) predicts suicide ideation. Suicide Life-Threatening Behav..

[15] DeCou CR, Schumann ME (2018). On the iatrogenic risk of assessing suicidality: A meta-analysis. Suicide Life-Threatening Behav..

[16] Blades CA, Stritzke WGK, Page AC, Brown JD (2018). The benefits and risks of asking research participants about suicide: A meta-analysis of the impact of exposure to suicide-related content. Clin. Psychol. Rev..

[17] Bender TW (2019). Does it hurt to ask? An analysis of iatrogenic risk during suicide risk assessment. Neurol. Psychiatry Brain Res..

[18] Cha CB (2016). Examining potential iatrogenic effects of viewing suicide and self-injury stimuli. Psychol. Assess..

[19] Mathias CW (2012). What’s the harm in asking about suicidal ideation?. Suicide Life-Threatening Behav..

[20] Franklin JC, Huang X, Bastidas D (2019). Virtual reality suicide: Development of a translational approach for studying suicide causes. Behav. Res. Ther..

[21] Huang X, Funsch KM, Park EC, Franklin JC (2020). Anticipated consequences as the primary causes of suicidal behavior: Evidence from a laboratory study. Behav. Res. Ther..

[22] Joiner TE, Walker RL, Rudd MD, Jobes DA (1999). Scientizing and routinizing the assessment of suicidality in outpatient practice. Prof. Psychol. Res. Pract..

[23] Chu C (2015). Routinized assessment of suicide risk in clinical practice: An empirically informed update. J. Clin. Psychol..

[24] Franklin JC (2017). Risk factors for suicidal thoughts and behaviors : A meta-analysis of 50 years of research. Psychol. Bull..

[25] Joiner, T. E. *Why People Die by Suicide*. (Harvard University Press, 2005).

[26] Van Orden KA (2010). The interpersonal theory of suicide. Psychol. Rev..

[27] American Association of Suicidology. Warning signs of acute suicide risk. https://suicidology.org/resources/warning-signs/ (2020).

[28] National Institute of Mental Health. Warning signs of suicide. https://www.nimh.nih.gov/health/publications/warning-signs-of-suicide/ (2020).

[29] Hatkevich C, Penner F, Sharp C (2019). Difficulties in emotion regulation and suicide ideation and attempt in adolescent inpatients. Psychiatry Res..

[30] Rajappa K, Gallagher M, Miranda R (2012). Emotion dysregulation and vulnerability to suicidal ideation and attempts. Cognit. Ther. Res..

[31] Fazaa N, Page S (2003). Dependency and self-criticism as predictors of suicidal behavior. Suicide Life-Threatening Behav..

[32] O’Connor RC, Noyce R (2008). Personality and cognitive processes: Self-criticism and different types of rumination as predictors of suicidal ideation. Behav. Res. Ther..

[33] Ribeiro JD (2014). Fearlessness about death: The psychometric properties and construct validity of the revision to the Acquired Capability for Suicide Scale. Psychol. Assess..

[34] Van Orden KA, Cukrowicz KC, Witte TK, Joiner TE (2012). Thwarted belongingness and perceived burdensomeness: Construct validity and psychometric properties of the Interpersonal Needs Questionnaire. Psychol. Assess..

[35] Ribeiro JD, Bender TW, Selby EA, Hames JL, Joiner TE (2011). Development and validation of a brief self-report measure of agitation: The Brief Agitation Measure. J. Pers. Assess..

[36] Gratz KL, Roemer L (2004). Multidimensional assessment of emotion regulation and dysregulation: Development, factor structure, and initial validation of the difficulties in emotion regulation scale. J. Psychopathol. Behav. Assess..

[37] Fox HC, Axelrod SR, Paliwal P, Sleeper J, Sinha R (2007). Difficulties in emotion regulation and impulse control during cocaine abstinence. Drug Alcohol Depend..

[38] Gratz KL, Tull MT, Baruch DE, Bornovalova MA, Lejuez CW (2008). Factors associated with co-occurring borderline personality disorder among inner-city substance users: the roles of childhood maltreatment, negative affect intensity/reactivity, and emotion dysregulation. Compr. Psychiatry.

[39] Johnson KA (2008). Linkages between cigarette smoking outcome expectancies and negative emotional vulnerability. Addict. Behav..

[40] Gratz KL, Rosenthal MZ, Tull MT, Lejuez CW, Gunderson JG (2006). An experimental investigation of emotion dysregulation in borderline personality disorder. J. Abnorm. Psychol..

[41] Gratz KL, Rosenthal MZ, Tull MT, Lejuez CW, Gunderson JG (2009). An experimental investigation of emotion dysregulation in borderline personality disorder. Personal. Disord. Theory.

[42] Hooley, J. M., Ho, D. T., Slater, J. & Lockshin, A. Pain perception and nonsuicidal self-injury: A laboratory investigation. *Personal. Disord. Theory, Res. Treat.* **1**, 170–179 (2010). 10.1037/a0020106 22448633

[43] Glassman LH, Weierich MR, Hooley JM, Deliberto TL, Nock MK (2007). Child maltreatment, non-suicidal self-injury, and the mediating role of self-criticism. Behav. Res. Ther..

[44] R Core Team. R: A language and environment for statistical computing. (2020).

[45] van Buuren, S. *et al.* Package ‘mice’: Multivariate imputation by chained equations. (2020).

[46] Derogatis LR, Melisaratos N (1983). The brief symptom inventory: an introductory report. Psychol. Med..

[47] Boulet J, Boss MW (1991). Reliability and validity of the brief symptom inventory. Psychol. Assess..

[48] Chu C (2017). The interpersonal theory of suicide: a systematic review and meta-analysis of a decade of cross-national research. Psychol. Bull..

[49] Huang, X., Ribeiro, J. D. & Franklin, J. C. The differences between suicide ideators and suicide attempters: Simple, complicated, or complex? *J. Consult. Clin. Psychol.* 1–16 (2020). 10.1037/ccp0000498 32105092

[50] Ribeiro JD, Huang X, Fox KR, Walsh CG, Linthicum KP (2019). Predicting imminent suicidal thoughts and nonfatal attempts: The role of complexity. Clin. Psychol. Sci..

[51] Ribeiro JD, Harris LL, Linthicum KP, Bryen CP (2020). Do suicidal behaviors increase the capability for suicide?: A longitudinal pretest-posttest study in over 1,000 high risk individuals. Clin. Psychol. Sci..

[52] Bryan CJ, Sinclair S, Heron EA (2016). Do military personnel ‘acquire’ the capability for suicide from combat?: A test of the interpersonal-psychological theory of suicide. Clin. Psychol. Sci..

[53] Dazzi T, Gribble R, Wessely S, Fear NT (2014). Does asking about suicide and related behaviours induce suicidal ideation? What is the evidence?. Psychol. Med..

[54] Hom MA (2018). Investigating the iatrogenic effects of repeated suicidal ideation screening on suicidal and depression symptoms: A staggered sequential study. J. Affect. Disord..

[55] Gould MS (2005). Evaluating iatrogenic risk of youth suicide screening programs: A randomized controlled trial. J. Am. Med. Assoc..

[56] Bellet BW, Jones PJ, McNally RJ (2018). Trigger warning: Empirical evidence ahead. J. Behav. Ther. Exp. Psychiatry.

[57] Jones PJ, Bellet BW, McNally RJ (2020). Helping or harming? The effect of trigger warnings on individuals with trauma histories. Clin. Psychol. Sci..

[58] Bridge JA (2020). Association between the release of Netflix’s 13 Reasons Why and suicide rates in the United States: An interrupted time series analysis. J. Am. Acad. Child Adolesc. Psychiatry.

[59] World Health Organization. *Preventing suicide: A resource for media professionals*. https://www.who.int/mental_health/prevention/suicide/resource_media.pdf (2017).

[60] American Foundation for Suicide Prevention. Reporting on suicide prevention. https://afsp.org/reporting-on-suicide-prevention (2021).

[61] Burke TA, Ammerman BA, Knorr AC, Alloy LB, Mccloskey MS (2018). Measuring acquired capability for suicide within an Ideation-to-Action Framework. Psychol. Violence.

