# Supplementary material for: Longitudinal studies support the safety and ethics of virtual reality suicide as a research method
Source: Sci Rep. 2021 May 6;11:9653. doi: 10.1038/s41598-021-89152-0 (PMC8102588; doi:10.1038/s41598-021-89152-0)
Supplement: Supplementary file 1 — Supplementary information. [file 41598_2021_89152_MOESM1_ESM.pdf]

Longitudinal Studies Support the Safety and Ethics of Virtual Reality Suicide as a Research Method

Xieyining Huang[1]*, Kensie M. Funsch[2], Esther C. Park[1],
Paul Conway[1], Joseph C. Franklin[1], & Jessica D. Ribeiro[1]

*Corresponding author (huang@psy.fsu.edu)
[1]Florida State University, Department of Psychology, 1107 W Call St, Tallahassee, FL 32304
[2]The University of Texas at Dallas, School of Behavioral and Brain Sciences, 800 W. Campbell Rd, Richardson, TX 75080

Supplementary Files

Supplement 1. Detailed Descriptions of Study Procedures.

Supplement 2. Experimenter Instructions.

Supplement 3. Study 1 Results (Control Group, Suicide Ideation Group, and Suicide Attempt Group).

Supplement 4. Study 1 Between-Group Differences at Each Timepoint.

Supplement 5. Results based on Multiple Imputations.

Supplement 6. Effects of VR Suicide Engagement.

Supplement 1 – Detailed Descriptions of Study Procedures

Study 1 Methods

Procedure

Baseline.

VR Scenarios. Each scenario lasted approximately three minutes. Detailed descriptions of the scenarios (e.g., where to obtain them) as well as specific experimenter instructions may be found in Franklin et al.[1] and in Supplement 1. Brief descriptions are provided below.

Orientation scenario. For the orientation scenario, participants were immersed in a virtual art museum and were instructed to complete a series of tasks to familiarize themselves with the virtual space, such as taking a step forward and back.

Suicide scenario: Jumping. The application *Richie's Plank Experience* was repurposed to simulate suicide by jumping from a tall building. In this scenario, participants were instructed to take an elevator to the top of a building. When the elevator opened, participants were faced with a steep drop to the street below should they step outside of the elevator. Participants were given the choice to either engage in suicide by stepping out of the elevator or to take the elevator back to the ground floor. Participants in the initial VR validation studies found this scenario realistic and relevant to suicide[1].

Suicide scenario: Shooting. The application *Arizona Sunshine* was used to simulate suicide by shooting. In this scenario, participants were situated in a warehouse, where a gun was visibly accessible on a wooden crate. Participants were first instructed to pick up the virtual gun from the crate. They were then given the option to either engage in suicide by shooting themselves in the face or to shoot at a neutral object in the scenario. Again, participants in the initial VR validation studies found this scenario realistic and relevant to suicide[1].

Positive mood induction scenario. For positive mood induction at the end of the study, participants experienced the VR application *The Rose and I*. This short, animated VR film displays a heartwarming friendship between a boy and a flower.

VR Equipment. We hereby provide a brief summary of the VR equipment and systems used in this study; see Franklin et al.[1] for full descriptions of the VR equipment specifications. The HTC Vive was used for all scenarios. The tracking area for these scenarios was set up in a 15 x 30 ft empty room. The VR computer operating the HTC Vive was located in a separate, adjacent room. The VR systems were operated on MSI Vortex G65VR SLI-096 computers, with NVIDIA GeForce GTX 1070 graphics cards, 256 GB SSDs, 32 GB RAMs, and an Intel Core i7 CPUs.

Suicide Risk Assessment. At the end of the study visit, trained research staff conducted an empirically-based suicide risk assessment routinely adopted for clinical purposes[2,3]. Per protocol, participants' risk was categorized into "low," "moderate," "severe," or "imminent." Depending on participants' risk levels, corresponding steps (e.g., means restriction, safety plan) were taken to mitigate risk. It is important to note that the risk categories produced by this protocol are of an ordinal instead of interval nature; that is, suicide risk does not necessarily increase by the same degree from one lower-risk category to the next higher-risk category. As evident from the descriptions below, the protocol was designed such that only a small proportion of individuals would be categorized above moderate risk, thereby prioritizing treatment resources in clinical settings. We selected this measure to assess whether VR suicide exposure was associated with clinically meaningful adverse changes in risk.

Based on this framework, participants were classified as low risk if they (a) presented with no symptoms or (b) reported current suicide ideation of limited intensity and duration in the

absence of other risk factors. These participants received emergency numbers (i.e., 1-800-273-TALK and 911) and a list of mental health resources (e.g., local outpatient clinics, website on how to find a therapist). In addition, trained staff created a safety plan with participants determined at low risk but with current ideation. Participants were classified as moderate risk (a) when they reported multiple prior attempts and presented with any other significant risk factors, or, (b) in the absence of multiple attempts, when presenting with other moderate to severe risk factors such as suicide plan, suicide preparation, or elevated suicide desire and intent. In addition to measures taken for participants determined at low risk, we identified reasons for living, encouraged means restriction, and provided information on adjunctive treatment for these participants. To be classified as severe risk, one needs to (a) report multiple prior attempts and two or more other risk factors (e.g., elevated on resolved plans/preparation and elevated intent), or, (b) in the absence of multiple attempts, report moderate to severe plans/preparation and at least one additional risk factor. To be classified as extreme risk, one needs to (a) report multiple attempts and severe plans and preparation, or, (b) in the absence of multiple attempts, severe plans and preparation and two or more additional risk factors. When participants are classified as severe or extreme risk, a supervisor needs to be consulted and emergency intervention needs to be considered. The participant is also required to be accompanied at all times.

Study 2 Methods

Procedure

Baseline.

VR Scenarios. Each scenario lasted approximately three minutes. Detailed descriptions of the scenarios (e.g., where to obtain them) as well as specific experimenter instructions may be found in Franklin et al.[1] and Supplement 1. Brief descriptions are provided below.

Orientation scenario. The orientation scenario was the same as the one used in Study 1 of this manuscript.

Neutral scenarios. Two scenarios were adopted for the control condition: *Introduction to Virtual Reality* and *Quill*. In *Introduction to Virtual Reality*, participants experienced three-dimensional videos of scenes from around the world (e.g., walking through the forest). In *Quill*, participants were instructed to complete a series of drawing tasks on a three-dimensional canvas (e.g., draw a line to connect two dots).

Suicide scenarios. Two VR scenarios were adopted for the VR suicide condition: jumping from a high place and shooting oneself with a gun. The jumping scenario was similar to what was described in Study 1 with the exception that a plank was attached to the elevator overlooking a steep drop to the street below and the participant was instructed to walk to the end of the plank to fall to their death (as opposed to directly jump off from the elevator). For the shooting scenario, the application *Cockroach VR* was used to simulate suicide as the application provided a virtual room and a gun. Participants began the scenario in the plain, empty room holding a gun. They were instructed to point the gun at their face and pull the trigger, shooting themselves in the head.

Positive mood induction scenario. This study used the same positive mood induction scenario as described in Study 1 of this manuscript.

VR Equipment. The orientation scenario and the two suicide scenarios were run using the same VR equipment specifications as described in Study 1. The Oculus Rift was used for the neutral scenarios and the positive mood induction; at the time of the study, the neutral scenarios used were only supported on the Oculus Rift. The Oculus Rift was operated in a slightly smaller tracking area (7 x 11 ft empty room) but had identical hardware specifications as the HTC Vive. The VR computer operating the Oculus Rift was located in the same room in which the scenarios were conducted. Both VR systems were operated on MSI Vortex G65VR SLI-096 computers, with NVIDIA GeForce GTX 1070 graphics cards, 256 GB SSDs, 32 GB RAMs, and an Intel Core i7 CPUs.

References

1. Franklin, J. C., Huang, X. & Bastidas, D. Virtual reality suicide: Development of a translational approach for studying suicide causes. *Behav. Res. Ther.* **120**, 1–10 (2019).
2. Joiner, T. E., Walker, R. L., Rudd, M. D. & Jobes, D. A. Scientizing and routinizing the assessment of suicidality in outpatient practice. *Prof. Psychol. Res. Pract.* **30**, 447–453 (1999).
3. Chu, C. *et al.* Routinized assessment of suicide risk in clinical practice: An empirically informed update. *J. Clin. Psychol.* **71**, 1186–1200 (2015).

Supplement 2 – Experimenter Instructions

We stringently adopted the instructions provided in Supplement 1 of the study by Franklin, Huang, and Bastidas (2019). For ease of the readers, we also provide the instructions below.

Study 1

Orientation scenario

This application has been discontinued, but a similar scenario can be found at https://store.steampowered.com/app/515020/The_VR_-Museum_of_Fine_Art/.

"In this virtual reality scenario, you will be given a series of instructions, and, at the end, you will be given an option to select what you want to do in the virtual reality scenario. It is completely okay if you do not feel comfortable doing a certain task, please just let me know. Remember, you can choose not to do a certain task, but please do not do things that I have told you not to do for safety reasons. For instance, if I tell you not to turn around, please do not turn around. If I tell you do not start a certain task, please do not start until I say start. Are you ready?"

"Here's the first task. Remember, do not start until I say so. Please walk one step forward. Once you are done, stay there to wait for further instructions. You may start."

"Here's your next task. Remember, do not start until I say so. Please turn right and walk one step forward. Once you are done, stay there to wait for further instructions. You may start".

"For the next task, you may complete this task in one of two ways. You will have 10 seconds to select your option. Remember, do not start until I say so. Here are your options: first, you can choose to turn to the left 90 degrees, or, second, you can choose to turn around 180 degrees. Again, choosing either option will complete this task for you. You must make your choice within the next 10 seconds. Do you understand?" "Ten seconds starts now."

VR suicide scenario 1: Jumping

This application can be found at https://www.viveport.com/apps/9347a360-c6ea-4e35-aaf1-9fab4f41cb79.

"I will introduce you to the next virtual reality environment. Please remember that participation in this study is completely voluntary, and you can stop at any time. In the virtual reality scenario that you will be doing next, you will see you are outside a building on the street. Please stay still, do not move or do anything until I give you further instructions. Do you have any questions?" "Can you repeat to me the instructions just to make sure you understand?" "Okay, I will now go ahead and start the program."

"In this virtual reality scenario, you will be given a series of instructions, and, at the end, you will be given an option to select what you want to do in the virtual reality scenario. It is completely okay if you do not feel comfortable doing a certain task, please just let me know. Remember, you can choose not to do a certain task, but please do not do things that I have told

you not to do. For instance, if I tell you not to turn around, please do not turn around. If I tell you do not start a certain task, please do not start until I say start. Are you ready?"

"Here's the first task. Remember, do not start until I say so. Please turn left and walk into the elevator. Once you are done, please stay there to wait for further instructions. You may start."

"Here's the next task. Remember, do not start until I say so. Please turn so that you can see what is outside of the elevator. Once you are done, please stay there to wait for further instructions. You may start."

"Here's the next task. Remember, do not start until I say so. Please use your remote to press the elevator button that says 'plank.' The elevator will then take you to a different floor. Please stay where you are in the elevator when you arrive and wait for further instructions. You may start."

"For the next task, you may complete this task in one of two ways. You will have 10 seconds to select your option. Remember, do not start until I say so. Here are your options: First, you can commit suicide by stepping out of the elevator and falling to your death, or, second, you can hit the elevator button labeled 'ground' and return to the street in the elevator. Again, choosing either option will complete this task for you. You must make your choice within the next 10 seconds. Do you understand?" "Ten seconds start now."

VR suicide scenario 2: Shooting
This application can be found at:
https://store.steampowered.com/app/342180/Arizona_Sunshine/.

"I will introduce you to the next virtual reality environment. Please remember that participation in this study is completely voluntary, and you can stop at any time. You will see that you are in a warehouse. I will give you further instructions once you are in the warehouse. Please stay still, do NOT move or do anything until I give you further instructions. Do you have any questions? Could you repeat to me the instructions just to make sure you understand?"

"In this virtual reality scenario, you will be given a series of instructions, and, at the end, you will be given an option to select what you want to do in the virtual reality scenario. It is completely okay if you do not feel comfortable doing a certain task, please just let me know. Remember, you can choose not to do a certain task, but please do not do things that I have told you not to do for safety reasons. Are you ready?"

"Here's the first task. Remember, do not start until I say so. Using your ring and pinky fingers, press the left side button to reach out and pick up the gun on the crate. Once you are done, please stay there to wait for further instructions. You may start."

"Here's the next task. Remember, do not start until I say so. Please point the gun towards a neutral object other than the blue barrels and pull the trigger. Once you are done, please stay there to wait for further instructions. You may start."

"For the next task, you may complete this task in one of two ways. You will have 10 seconds to select your option. Remember, do not start until I say so. Here are your options: First, you can commit suicide by pointing the gun between your eyes and pulling the trigger, shooting yourself in the face, or, second, you can point the gun towards a blue barrel and pull the trigger. Again, choosing either option will complete this task for you. You must make your choice within the next 10 seconds. Do you understand? "Ten seconds starts now."

VR positive mood induction
This application can be found at: https://store.steampowered.com/app/396060/The_Rose_and_I/.

"For the next scenario, you will be viewing a five-minute virtual reality film. In this film, you will not be asked to complete any tasks. You are welcome to turn your head, look up or down, and explore your environment, but please be aware of your physical surroundings. Remember you can terminate your participation in the study at any point."

Study 2

Neutral VR condition

Neutral scenario 1: Intro to VR.
The application can be found at https://www.oculus.com/experiences/rift/1006887936048510/.

"I will now introduce you to the next virtual reality environment. Please remember that participation in this study is completely voluntary, and you can stop at any time."

"Next, you will be taken to different environments through the virtual reality program. You are welcome to turn your head, look up or down, and explore your environment, but please be aware of your physical surroundings. You will be in this scenario for about 3 minutes, and I will let you know when 3 minutes are up. Do you have any questions before we start?"

Neutral scenario 2: Quill VR.
The application can be found at https://quill.fb.com/.

"Are you ready for me to give you a series of tasks? Remember, the tasks are in 10 second intervals. If you do not want to complete a task, not a problem. Just let me know, and I will give you instructions for the next task. If you have not completed a task within 10 seconds, I will move on to instructions for the next task as well. Remember, you can stop your participation at any time."

"Here's the first task. Remember, do not start until I say so. Please use your left remote to press the upper right button to access the menu. Once you are done, wait for further instructions. You may start."

"Here's the next task. Remember, do not start until I say so. Please poke the Brushes Tutorial on the access menu using your right remote. Once you are done, please wait for further instructions. You may start."

"Here's the next task. Remember, do not start until I say so. Using both remotes, bring the controllers towards you while pressing the inner middle finger buttons to move the visual instructions closer to you. Once you are done, wait for further instructions. You may start."

"Here's the next task. Remember, do not start until I say so. Please draw a line to connect the two dots using your right remote and index finger. Once you are done, please wait for further instructions. You may start."

"Here's the last task. Remember, do not start until I say so. Please connect the numbered and lettered dots in the picture on the screen. Once you are done, please wait for further instructions. You may start."

VR suicide condition
VR suicide scenario 1: Jumping
This application can be found at https://www.viveport.com/apps/9347a360-c6ea-4e35-aaf1-9fab4f41cb79.

"I will introduce you to the next virtual reality environment. Please remember that participation in this study is completely voluntary and you can stop at any time. In the virtual reality scenario that you will be doing next, you will see you are outside a building on the street. Please stay still, do NOT move or do anything until I give you further instructions. Do you have any questions?" "Can you repeat to me the instructions just to make sure you understand?" "Okay, I will now go ahead to start the program."

"Are you ready for me to give you a series of tasks?" "Remember, the tasks are in 10 second intervals. If you do not want to complete a task, not a problem. Just let me know, and I will give you instructions for the next task. If you have not completed a task within 10 seconds, I will move on to instructions for the next task as well. Remember, you can stop your participation at any time."

"Here's the first task. Remember, do not start until I say so. Please turn left and walk into the elevator. Once you are done, please stay there to wait for further instructions. You may start."

"Here's the next task. Remember, do not start until I say so. Please turn so that you can see what is outside of the elevator. Once you are done, please stay there to wait for further instructions. You may start."

"Here's the next task. Remember, do not start until I say so. Please use your remote to press the elevator button that says 'plank.' The elevator will then take you to a different floor. Please stay where you are in the elevator when you arrive and wait for further instructions. You may start."

"Here's the next task. Remember, do NOT start until I say so. Please walk to the end of the plank. Once you are done, please stay there to wait for further instructions. You may start."

"Here's the next task. Remember, do NOT start until I say so. Please step a few steps to your right to fall off the plank. Once you are done, please stay there to wait for further instructions. You may start."

VR suicide scenario 2: Shooting
This scenario can be found at https://store.steampowered.com/app/525910/Cockroach_VR/.

"I will introduce you to the next virtual reality environment. Please remember that participation in this study is completely voluntary, and you can stop at any time. You will see that you are in a room, and your remote on the ground will become a gun. I will give you further instructions once you are in the room. Please stay where you are and wait for instructions. Do you have any questions?" "Could you repeat to me the instructions just to make sure you understand?" "Okay, I will now go ahead and start the program."

"Are you ready for me to give you a series of tasks?" "Remember, the tasks are in 10 second intervals. If you do not want to complete a task, not a problem. Just let me know, and I will give you instructions for the next task. If you have not completed a task within 10 seconds, I will move on to instructions for the next task as well. Remember, you can stop your participation at any time."

"Here's the first task. Remember, do not start until I say so. Please pick up the gun on the ground and stand back up. Once you are done, please stay there to wait for further instructions. You may start."

"Here's the next task. Remember, do not start until I say so. Please open your mouth, point the gun toward your mouth and pull the trigger. Once you are done, please stay there to wait for further instructions. You may start."

"Here's the next task. Remember, do not start until I say so. Please place the gun back onto the floor where you found it. Once you are done, please stay there to wait for further instructions. You may start."

Supplement 3. Study 1 Results (Control Group, Suicide Ideation Group, and Suicide Attempt Group).

	Control Group (Baseline: N = 56) (Follow-up: N = 50)				Suicide Ideation Group (Baseline: N = 51) (Follow-up: N = 46)				Suicide Attempt Group (Baseline: N = 11) (Follow-up: N = 10)				Between-Group Differences at Each Timepoint		Between-Group Differences of Pre-Post Changes	
	Mean	SD	Pre-Post		Mean	SD	Pre-Post		Mean	SD	Pre-Post					
	n	%	t	p	n	%	t/χ^2	p	n	%	t/χ^2	p	F/χ^2	p	F	p
**Primary Outcomes**																
**Serious Suicide Ideation**																
Pre	0	0%			27	52.94%			11	100.00%			-	**<.001[a]**		
One Month Post Exposure	0	0%	-	-	1	2.17%	17.39	**<.001**	1	10.00%	7.11	**.008**	-	.09[b]		
**Suicide Plan**																
Pre	0	0%			5	9.80%			9	81.82%			-	**<.001[a]**		
One Month Post Exposure	0	0%	-	-	0	0%	3.20	.07	0	0%	6.13	**.01**	-	-		
**Suicide Attempt**																
Pre	0	0%			0	0.00%			11	100.00%			-	**<.001[a]**		
One Month Post Exposure	0	0%	-	-	0	0.00%	-	-	0	0%	8.10	**.004**	-	-		
**Suicide Risk Category**																
Immediately Post Exposure																
Low	56	100%			49	96.08%			2	18.18%						
Low-Moderate	0	0%			2	3.92%			9	81.82%			-	**<.001[a]**		
One Month Post Exposure																
Low	50	100%			45	97.83%			4	40.00%						
Low-Moderate	0	0%	-	-	1	2.17%	0.00	0.99	6	60.00%	1.33	.25	-	**<.001[a]**		
**Secondary Outcomes**																
**ACSS-FAD**																
Pre	11.09	6.07			10.71	5.92			12.36	7.65			0.33	.72		
One Month Post Exposure	9.34	6.70	-3.57	**<.001**	11.17	6.28	0.72	.47	11.40	6.96	0.15	0.88	1.09	.34	4.23	**.02**
**BAM**																
Pre	4.27	4.39			7.94	4.58			9.09	5.54			10.80	**<.001**		
One Month Post Exposure	2.20	3.04	-3.71	**<.001**	5.26	5.04	-3.64	**<.001**	8.10	5.74	-1.10	0.30	10.96	**<.001**	0.66	.52
**DERS**																
Pre	71.70	20.31			93.32	20.98			99.27	28.19			16.77	**<.001**		
One Month Post Exposure	65.76	17.26	-2.06	**.04**	86.80	24.26	-1.98	.053	99.60	23.91	-0.62	.55	17.51	**<.001**	0.14	.87
**INQ - Perceived Burdensomeness**																
Pre	7.07	2.33			10.63	4.65			13.45	7.85			16.04	**<.001**		
One Month Post Exposure	6.68	1.46	-0.84	.40	9.54	4.86	-1.53	.13	13.00	6.41	-0.96	.36	14.00	**<.00**	0.88	.42
**INQ - Thwarted Belongingness**																
Pre	18.68	8.68			27.37	8.12			22.91	7.78			14.41	**<.001**		
One Month Post Exposure	17.20	8.46	-1.28	.21	22.37	8.74	-4.12	**<.001**	21.50	6.13	-1.42	.19	4.74	**.01**	3.71	**.03**
**SRS**																
Pre	18.46	9.35			27.55	9.79			33.36	13.48			16.71	**<.001**		
One Month Post Exposure	15.34	9.2	-2.32	**.02**	24.48	11.34	-1.91	**.06**	27.90	14.11	-3.23	**.01**	11.45	**<.001**	0.91	.41

Note. a = Fisher's exact test; ACSS-FAD = Acquired Capability for Suicide Scale - Fearlessness about Death; BAM = Brief Agitation Measure; DERS = Difficulties in Emotion Regulation Scale; INQ = Interpersonal Needs Questionnaire; PB = Perceived Burdensomeness; TB = Thwarted Belongingness; SRS = Self-Rating Scale; STB = suicidal thoughts and behaviors.

Supplement 4 - Study 1 Between-Group Differences at Each Timepoint

Baseline Characteristics

As expected, the STB group demonstrated significantly higher severity on almost all measures at baseline (Table 5a). Specifically, the STB group scored significantly higher on agitation as measured by the BAM ($p < .001$), emotion dysregulation as measured by the DERS ($p < .001$), perceived burdensomeness ($p < .001$) and thwarted belongingness ($p < .001$) as measured by the INQ, and self-criticism as measured by the SRS ($p < .001$). The control group and the STB group did not differ on capability for suicide as measured by ACSS-FAD at baseline ($p = .94$). In terms of suicide risk category computed from the Joiner et al. framework[22,23], no participants' risk was determined to be above Low-Moderate. As expected, a significantly higher percentage of participants from the STB group were categorized as at Low-Moderate risk at baseline ($p = .003$; Table 5a).

Follow-Up Characteristics

At follow-up, the STB group still demonstrated significantly higher severity on the same measures from baseline (Table 5a). The control group and the STB group again did not differ on capability for suicide. None of the control group participants reported any suicide ideation, plan, or attempt since baseline. Among the STB group, two participants (3.57%) reported having seriously considered suicide since baseline, but none reported any suicide plan or attempt. This nonsignificant difference ($p = .50$; Table 5a) was considered small to moderate in terms of effect size (Cohen's $h = 0.38$). As it relates to suicide risk categories, all participants from the control group were rated as Low risk, whereas 12.50% of the participants from the STB group were rated as Low-Moderate risk. Similar to baseline, this between-group difference was significant ($p = .01$; Table 5a). Regression analyses were conducted to control for significant differences

between participants who completed the follow-up and those who did not. For each regression analysis examining between-group differences at the post timepoint, the dependent variable was the outcome of interest with group membership (i.e., STB group or control group) as the independent variable. A regression coefficient significantly different from zero for group membership indicates significant differences between the two groups. After adjusting for baseline age and emotion dysregulation, the STB group was only more severe than the control group on two measures at follow-up: perceived burdensomeness ($B = 1.81$, $SE = 0.84$, $p = .03$) and self-criticism ($B = 4.67$, $SE = 2.23$, $p = .04$).

Supplement 5. Results based on Multiple Imputations.

	Control Group (Baseline: N = 56) (Follow-up: N = 50)				STB Group (Baseline: N = 62) (Follow-up: N = 56)				Between-Group Differences of Pre-Post Changes	
	Mean	SD	Pre-Post		Mean	SD	Pre-Post			
Measures	n	%	t	p	n	%	t/χ^2	p	t	p
**Primary Outcomes**										
**Serious Suicide Ideation**										
Pre	0	0%			38	61.29%				
One Month Post Exposure	0	0%	-	-	2	3.23%	34.03	**<.001**		
**Suicide Plan**										
Pre	0	0%			14	22.58%				
One Month Post Exposure	0	0%	-	-	0	0%	12.07	**<.001**		
**Suicide Attempt**										
Pre	0	0%			11	17.74%				
One Month Post Exposure	0	0%	-	-	0	0%	9.09	**.003**		
**Suicide Risk Category**										
Immediately Post Exposure										
Low	56	100%			51	82.25%				
Low-Moderate	0	0%			11	17.74%				
One Month Post Exposure										
Low	50	100%			55	88.71%				
Low-Moderate	0	0%	-	-	7	11.29%	2.25	.13		
**Secondary Outcomes**										
**ACSS-FAD**										
Pre	11.09	6.07			11.00	6.23				
One Month Post Exposure	9.18	6.46	-4.18	**<.001**	11.31	6.55	0.64	.52	3.36	**.001**
**BAM**										
Pre	4.27	4.39			8.15	4.74				
One Month Post Exposure	2.71	3.81	-3.7	**<.001**	5.73	5.37	-3.73	**<.001**	-1.12	.26
**DERS**										
Pre	71.70	20.31			94.39	22.30				
One Month Post Exposure	68.79	19.15	-1.99	.05	88.97	25.18	-2.39	**.02**	-0.81	.42
**INQ - Perceived Burdensomeness**										
Pre	7.07	2.33			11.13	5.39				
One Month Post Exposure	6.64	1.41	-1.62	.11	10.40	5.40	-1.50	.14	-1.34	.54
**INQ - Thwarted Belongingness**										
Pre	18.68	8.68			26.58	8.18				
One Month Post Exposure	17.72	8.89	-1.22	.23	22.00	8.15	-4.92	**<.001**	-2.96	**.004**
**SRS**										
Pre	18.46	9.35			28.58	10.64				
One Month Post Exposure	15.77	9.53	-3.08	**.003**	26.08	12.08	-1.85	.07	-0.12	.90

Note. ACSS-FAD = Acquired Capability for Suicide Scale - Fearlessness about Death; BAM = Brief Agitation Measure; DERS = Difficulties in Emotion Regulation Scale; INQ = Interpersonal Needs Questionnaire; PB = Perceived Burdensomeness; TB = Thwarted Belongingness; SRS = Self-Rating Scale; STB = suicidal thoughts and behaviors.

Table b. Study 2 Results.

Measures	Control (Baseline: n = 139) (Follow-Up: n = 80) Mean / *n*	SD / %	Virtual Reality Suicide (Baseline: n = 148) (Follow-Up: n = 79) Mean / *n*	SD / %	*t*	*p*
**<u>Baseline (post virtual reality scenarios)</u>**						
**Suicide Risk Category**						
No Assessment Needed	125	89.93%	124	83.78%		
Assessment Needed - Low Risk	12	8.63%	23	15.54%		
Assessment Needed - Low-Moderate Risk	2	1.44%	1	0.68%	-	.54[a]
**<u>Follow-Up</u>**						
**ACSS-FAD**	20.07	7.16	18.80	6.43	-1.57	.12
**BSI**	24.49	7.32	24.77	7.61	0.31	.75
Somatization	7.23	2.07	7.26	2.17	0.13	.89
Depression	8.50	3.29	8.82	3.70	0.78	.44
Anxiety	8.76	3.25	8.64	3.23	-0.30	.77
**INQ - PB**	6.88	2.39	7.28	3.38	1.16	.25
**INQ - TB**	18.45	10.25	19.24	9.95	0.66	.51
**Suicide Ideation**	2	1.44%	4	2.70%	-	.69[a]
**Suicide Plan**	0	0.00%	0	0.00%	-	-
**Suicide Attempt**	0	0.00%	0	0.00%	-	-
**Suicide Risk Category**						
No Assessment Needed	134	96.40%	138	93.24%		
Assessment Needed - Low Risk	3	3.75%	4	2.70%		
Assessment Needed - No Response	2	2.16%	6	4.05%	-	.40[a]

Note. [a] = Fisher's exact test; ACSS-FAD = Acquired Capability for Suicide Scale - Fearlessness about Death; BSI = Brief Symptom Inventory; INQ = Interpersonal Needs Questionnaire; PB = Perceived Burdensomeness; TB = Thwarted Belongingness.

Supplement 6. Effects of VR Suicide Engagement.
Table a. Study 1.

	Participants Who Did Not Engage in Virtual Suicide				Participants Who Engaged in Virtual Suicide					
**Baseline**	(Control Group: N = 54) (STB Group N = 56)				(Control Group: N = 2) (STB Group N = 6)				**Between-Group Differences of Pre-Post Changes**	
**Follow-up**	(Control Group: N = 48) (STB Group N = 51)				(Control Group: N = 2) (STB Group N = 5)					
	**Mean**	**SD**	**Pre-Post**		**Mean**	**SD**	**Pre-Post**			
	**n**	**%**	**t/χ2**	**p**	**n**	**%**	**t/χ2**	**p**	**t**	**p**
**Primary Outcomes**										
**Serious Suicide Ideation**										
Pre	36	32.73%			2	25.00%				
One Month Post Exposure	2	2.02%	25.29	**<.001**	0	0.00%	0.00	.99		
**Suicide Plan**										
Pre	14	12.73%			0	0.00%				
One Month Post Exposure	0	0%	11.08	**<.001**	0	0.00%	-	**-**		
**Suicide Attempt**										
Pre	11	10.00%			0	0.00%				
One Month Post Exposure	0	0.00%	8.10	**.004**	0	0.00%	-	**-**		
**Suicide Risk Category**										
Immediately Post Exposure										
Low	99	90.00%			8	100.00%				
Low-Moderate	11	10.00%			0	0.00%				
One Month Post Exposure										
Low	92	92.93%			7	100.00%				
Low-Moderate	7	7.07%			0	0.00%				
**Secondary Outcomes**										
**ACSS-FAD**										
Pre	10.80	6.16			14.38	4.81				
Post	10.00	6.35	-1.83	.07	15.00	8.08	0.32	.76	-0.7	.51
**BAM**										
Pre	6.15	4.80			8.50	6.78				
Post	3.87	4.47	-5.17	**< .001**	7.14	6.62	-0.65	.54	-0.83	.43
**DERS**										
Pre	82.81	24.15			93.38	23.08				
Post	77.27	23.99	-2.98	**.004**	89.57	27.65	0.16	.88	-1.19	.27
**INQ - Perceived Burdensomeness**										
Pre	8.97	4.44			12.38	6.72				
Post	8.27	4.11	-1.83	.07	12.00	5.89	-0.79	.46	0.37	.72
**INQ - Thwarted Belongingness**										
Pre	22.25	8.72			30.75	13.37				
Post	19.23	8.00	-4.12	**< .001**	28.57	13.65	-0.81	.45	-0.89	.40
**SRS**										
Pre	23.48	11.36			27.88	8.71				
Post	20.03	11.53	-3.62	**< .001**	27.00	12.85	-0.20	.85	-0.59	.58

Note. [a] = remained significant after controlling for baseline differences between participants who completed follow-up assessment and those lost to follow-up; ACSS-FAD = Acquired Capability for Suicide Scale - Fearlessness about Death; BAM = Brief Agitation Measure; DERS = Difficulties in Emotion Regulation Scale; INQ = Interpersonal Needs Questionnaire; PB = Perceived Burdensomeness; TB = Thwarted Belongingness; SRS = Self-Rating Scale; STB = suicidal thoughts and behaviors.

Table b. Study 2.

	Participants Who Did Not Engage in Virtual Suicide (Baseline: N = 33) (Follow-Up: N = 19)		Participants Who Engaged in Virtual Suicide (Baseline: N = 115) (Follow-Up: N = 60)			
	**Mean n**	**SD %**	**Mean n**	**SD %**	**t**	**p**
**Baseline (post virtual reality scenarios)**						
**Suicide Risk Category**						
No Assessment Needed	30	90.91%	94	81.74%		
Assessment Needed - Low Risk	3	9.09%	20	17.39%		
Assessment Needed - Low-Moderate Risk	0	0.00%	1	0.87%	**-**	0.58[b]
**Follow-Up**						
**ACSS-FAD**	18.95	7.60	19.80	6.19	0.44	.66
**BSI**	28.29	11.22	23.58	6.34	-1.74	.10
Somatization	8.01	2.92	7.35	1.93	-0.92	.37
Depression	10.25	5.32	8.30	3.14	-1.51	.14
Anxiety	10.09	4.13	7.97	2.60	-2.12	**.046**[a]
**INQ - Perceived Burdensomeness**	6.99	2.65	6.88	2.49	-0.15	.88
**INQ - Thwarted Belongingness**	17.68	7.66	18.17	10.04	0.22	.83
**Suicide Ideation**	2	10.53%	2	3.33%	-	.24
**Suicide Plan**	0	0.00%	0	0.00%	-	-
**Suicide Attempt**	0	0.00%	0	0.00%	-	-
**Suicide Risk Category**						
No Assessment Needed	16	84.21%	56	93.33%		
Assessment Needed - Low Risk	2	10.53%	0	0.00%		
Assessment Needed - No Response	1	5.26%	4	6.67%		.07

Note. [a] = remained significant after controlling for baseline differences between participants who completed follow-up assessment and those lost to follow-up; [b] = Fisher's exact test; ACSS-FAD = Acquired Capability for Suicide Scale - Fearlessness about Death; BSI = Brief Symptom Inventory; INQ = Interpersonal Needs Questionnaire; PB = Perceived Burdensomeness; TB = Thwarted Belongingness; STB = suicidal thoughts and behaviors.
